# Seasonality and Genotype Diversity of Human Rhinoviruses during an Eight-Year Period in Slovenia

**DOI:** 10.3390/microorganisms12020341

**Published:** 2024-02-06

**Authors:** Nataša Berginc, Maja Sočan, Katarina Prosenc Trilar, Miroslav Petrovec

**Affiliations:** 1 Department of Public Health Microbiology, National Laboratory of Health, Environment and Food, 1000 Ljubljana, Slovenia; katarina.prosenc.trilar@nlzoh.si; 2 Centre for Infectious Diseases, National Institute of Public Health, 1000 Ljubljana, Slovenia; 3 Institute of Microbiology and Immunology, Faculty of Medicine, University of Ljubljana, 1000 Ljubljana, Slovenia

**Keywords:** rhinovirus, infection, seasonality, genotype diversity, demographic factors, meteorological factors, molecular epidemiology

## Abstract

Due to the high socioeconomic burden of rhinoviruses, the development of prevention and treatment strategies is of high importance. Understanding the epidemiological and clinical features of rhinoviruses is essential in order to address these issues. Our study aimed to define the seasonality and molecular epidemiology of rhinoviruses in Slovenia. Over a period of eight years, a total of 20,425 patients from sentinel primary healthcare settings and sentinel hospitals were examined for a panel of respiratory viruses in the national programme for the surveillance of influenza-like illnesses and acute respiratory infections. The patients were from all age groups and had respiratory infections of various severity. Infection with a rhinovirus was confirmed using an RT-rPCR in 1834 patients, and 1480 rhinoviruses were genotyped. The molecular analysis was linked to demographical and meteorological data. We confirmed the year-round circulation of rhinoviruses with clear seasonal cycles, resulting in two seasonal waves with peaks in spring and autumn. High levels of genotype variability and co-circulation were confirmed between and within seasons and were analysed in terms of patient age, the patient source reflecting disease severity, and meteorological factors. Our study provides missing scientific information on the genotype diversity of rhinoviruses in Slovenia. As most previous investigations focused on exclusive segments of the population, such as children or hospitalised patients, and for shorter study periods, our study, with its design, size and length, contributes complementary aspects and new evidence-based knowledge to the regional and global understanding of rhinovirus seasonality and molecular epidemiology.

## 1. Introduction

It has been well established that rhinoviruses (RVs) are one of the most common causative agents of respiratory infections in humans, with a high socioeconomic burden [1,2,3,4]. First discovered in the 1950s, these ubiquitous viruses are responsible for more than 50% of common cold episodes yearly [5]. Furthermore, during the last two decades, the increased implementation of multiplex molecular diagnostic methods for the rapid detection of respiratory viruses has facilitated the confirmation of RVs as causative agents of more severe lower respiratory tract infections in hospitalised patients, enhancing their public health relevance [4,5]. The treatment of RV infections remains primarily supportive, but given the frequency of these infections and the expanding knowledge of their broad clinical spectrum, effective control through prevention and treatment could have a significant public health benefit [5].

Rhinoviruses belong to the genus *Enterovirus* within the family *Picornaviridae* and form a heterogeneous group consisting of three species, namely, RV-A, RV-B and RV-C, which, to date, comprise the following 169 genotypes: 80 types of RV-A, 32 types of RV-B and 57 types of RV-C [5,6,7,8]. Rhinoviruses are non-enveloped RNA viruses with a positive-sense, single-stranded genome of approximately 7000 nucleotides, encoding four structural proteins (VP1, VP2, VP3 and VP4) and seven non-structural proteins [5,6,9].

Genetic relationships form the basis of the current RV taxonomy [8,10]. In contrast to some other members of the *Picornaviridae* family, a lack of capsid recombination was reported to be characteristic of RVs [10,11]. Consequently, the VP1 and VP4/VP2 genomic regions were both considered suitable for classification, but since the VP1 genomic region shows the highest precision, it currently represents the basis for RV genotype assignments, as it does for the group of enteroviruses from the same *Enterovirus* genus [6,10]. However, because it was demonstrated that RV types are grouped congruently based on the analysis of VP1 or VP4/VP2 genomic regions and because, in comparison to the VP1 genomic region, the analysis of the VP4/VP2 genomic region is facilitated by the ability to use a single amplification protocol, including primers, to genotype all three species (RV-A, RV-B and RV-C), the analysis of the VP4/VP2 genomic region is most often used in epidemiological and clinical studies of RVs [10,11].

The virological, epidemiological and clinical aspects of RV infections have been investigated in studies from all continents [4,5]. However, despite these efforts, it remains difficult to make comparisons and unequivocal links between RV species or types with clinical manifestations and disease severity or seasonality [4]. A large proportion of studies focused on specific segments of the population (children, hospitalised patients, etc.) or were limited to relatively short investigation periods (on average, between 1 and 3 years); furthermore, different studies often have inconsistent or even contrasting results on RV seasonality and the clinical aspects of RV infections [1,3,4,5,6,12,13,14,15,16,17,18].

Our large-scale systematic analysis of RVs’ molecular epidemiology aimed to provide missing scientific information on RVs circulating through an extended period of eight years, including patients from all age groups that were examined in primary healthcare settings and in hospitals. Additionally, the impact of meteorological factors on RV’s circulation was studied. Due to its size, length and study design, our investigation adds complementary aspects and contributes new evidence-based knowledge to the regional and global understanding of RV seasonality and molecular epidemiology.

## 2. Materials and Methods

### 2.1. Study Design and Period

We conducted a prospective study from week 40 in 2011 to week 39 in 2019 (a total of eight respiratory virus seasons). A respiratory virus season was defined from week 40 of a given calendar year up to week 39 of the next calendar year.

### 2.2. Sample Collection and Data Source

Combined nasal and throat swabs, together with standardised demographic and clinical data, from patients with an influenza-like illness (ILI) or acute respiratory infection (ARI) were collected in the national sentinel programme for ILI and ARI surveillance, which was conducted jointly by the National Laboratory of Health, Environment and Food and the National Institute of Public Health [19]. The sentinel comprises 50 primary healthcare clinics from all Slovenian regions, which also collect data on Medically Attended Acute Respiratory Illnesses (MAARIs), and two sentinel hospitals include patients with Severe Acute Respiratory Infections (SARIs). In this study, the patients examined at primary healthcare clinics (patients with MAARI) were considered cases of milder disease, and patients examined at hospitals (patients with SARI) were considered cases of severe disease. Patients with respiratory infections were sampled and tested for a panel of respiratory viruses, including RVs. The patients positive for RVs were included in this study.

Meteorological data (average temperature and average relative humidity) were collected from the database of the National Meteorological Services at the Slovenian Environmental Agency [20].

### 2.3. Molecular Identification of Rhinoviruses

Nucleic acids were extracted from 200 µL of combined nasal and throat swabs using membrane extraction kits following the manufacturer’s instructions (QIAamp MinElute Virus Spin Kit, 57704, or QIAamp 96 Virus QIAcubeHT, 57731) and stored at −70 °C.

The samples were then tested for a panel of respiratory viruses in a series of multiplex in-house reverse transcription real-time polymerase chain reactions (RT-rPCRs), as previously described [21,22,23,24,25,26,27,28]. Specifically, the presence of RV nucleic acids in the samples was detected by an RT-rPCR using a set of primers and a probe targeting the specific RV 5′UTR, as described by Auburn and colleagues [24].

### 2.4. Genotyping of Rhinoviruses

In the following steps, nucleotide sequences for genotyping RVs were obtained via Sanger sequencing, as described below. In summary, PCR amplicons were obtained, checked by gel electrophoresis, and purified with the QIAquick PCR Purification Kit, 28104, or QIAquick Gel Extraction Kit, 28704, following the manufacturer’s instructions. Mixes of adequately concentrated purified PCR amplicons and sequencing primers were prepared as per the instructions and shipped to the outsourced service provider for Sanger sequencing using their standard protocol (Eurofins Genomics, Ebersberg, Germany). Bioedit was used for contig trimming and assembly into RV partial VP4/VP2 or partial VP1 nucleotide sequences [29].

For RV genotyping, a partial VP4/VP2 genomic region was used. For all samples positive for RVs, part of the VP4/VP2 gene was amplified using a PCR, as described by Wisdom and colleagues, generating an amplicon of up to 540 nucleotides in length [11]. The identification of RV types was performed by comparing their partial VP4/VP2 genomic sequence to the panel of RV-type reference strains defined by the *Picornaviridae* Study Group at ICTV [7] and some additional strains used in previous studies [3,12]. All reference sequences were retrieved from NCBI GenBank [30]. Phylogenetic comparisons were performed in Nucleotide BLAST using the Megablast algorithm [30]. The criteria for RV-type identification defined by McIntire, Simmonds and colleagues were used, namely, over 10% divergence in nucleotides compared to the reference strains between types of RV-A and RV-C and over 9.5% divergence in nucleotides compared to the reference strains between types of RV-B [10].

For a very limited number of RVs, genotyping based on the VP4/VP2 genomic region was not successful, so part of the VP1 gene was additionally amplified by a PCR, as described by Nix, Oberste and colleagues or by Mubareka and colleagues [31,32]. The identification of RV types was performed by comparing the partial VP1 genomic sequence to the panel of RV-type reference strains defined by the *Picornaviridae* Study Group at ICTV [7]. All reference sequences were retrieved from GenBank [30]. Phylogenetic comparisons were performed in Nucleotide BLAST using the Megablast algorithm [30]. The criteria for RV-type identification defined by McIntire, Simmonds and colleagues were used, namely, over 13% divergence in nucleotides compared to reference strains between types of RV-A and RV-C and over 12% divergence in nucleotides compared to reference strains between types of RV-B [10].

For a very limited number of cases, when the genotyping described above based on the VP4/VP2 or VP1 genomic region was not successful, the determination of type was attempted by means of a phylogenetic tree construction using MEGA 7 [33]. Nucleotide sequences were aligned using ClustalW and alignments with a p-distance lower than 0.8 (Overall Mean Distance) were used for phylogenetic tree construction with the Neighbour-Joining algorithm and the Kimura 2-parameter model, with 1000 bootstrap replicates [34]. In these cases, the RV type was assigned based on the nearest reference strain in the phylogenetic tree. The phylogenetic trees were based on the RV-type reference strains defined by the *Picornaviridae* Study Group at ICTV, and enterovirus EV-D68 (GenBank ID: OP321154.1) was used as the outgroup [3,7]. All reference sequences were retrieved from GenBank [30].

### 2.5. Statistical Analyses

The data were analysed, and the results were illustrated using tools in Microsoft Office. All statistical analyses were performed with the open-source statistical software JASP [35]. A *p*-value of <0.05 was considered statistically significant. For the correlation analyses, a Chi-square test, Fischer’s exact test or Pearson’s correlation coefficient were calculated according to the investigated datasets. Comparisons between groups were performed using Student’s *t*-test, the Mann–Whitney U test or ANOVA according to the investigated datasets. The seasonal circulation of RVs was analysed using generalised linear modelling, and the effect of meteorological factors on RV circulation was analysed using multiple regression.

### 2.6. Ethical Considerations

This study was approved by the Committee for Medical Ethics of the Republic of Slovenia (No. 0120-206/2019/5).

## 3. Results

### 3.1. Molecular Identification and Genotyping of Rhinoviruses

Over a study period of eight years (eight respiratory virus seasons), a total of 20,425 patients with MAARI and SARI were tested with an RT-rPCR for a panel of respiratory viruses, including RVs, and 1834 (9%) of them were positive for RVs [21,22,23,24,25,26,27,28]. The distribution of RV-positive patients by age, gender and patient source is shown in Table 1.

Out of 1834 RV-positive samples, 1480 RVs (81%) were successfully genotyped; for 45 RVs (2%), only the RV species was successfully determined, and 309 RVs (17%) were not successfully genotyped. The most predominant species was RV-A, followed by RV-C and RV-B (59%, 38% and 3%, respectively).

**Figure 1:**
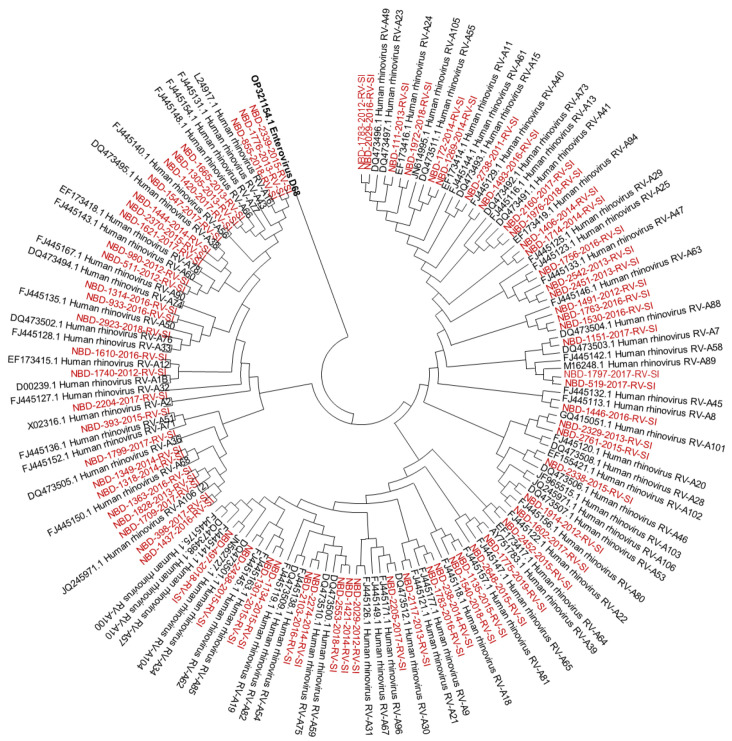
Phylogenetic tree (RV-A) constructed using the Neighbour-Joining algorithm, based on the VP4/VP2 region of RVs, including the selection of RVs genotyped in this study and RV-type reference strains defined by the *Picornaviridae* Study Group at ICTV [7]. Enterovirus EV-D68 (GenBank accession number: OP321154.1) was used as the outgroup. The RVs genotyped in this study are shown in red and labelled by the identifier NBD, followed by the laboratory protocol number, the collection year and the identifier RV-SI (NBD-PPPP-YYYY-RV-SI). All reference strains were retrieved from GenBank and are labelled by their GenBank accession numbers and RV type [30].

**Figure 2:**
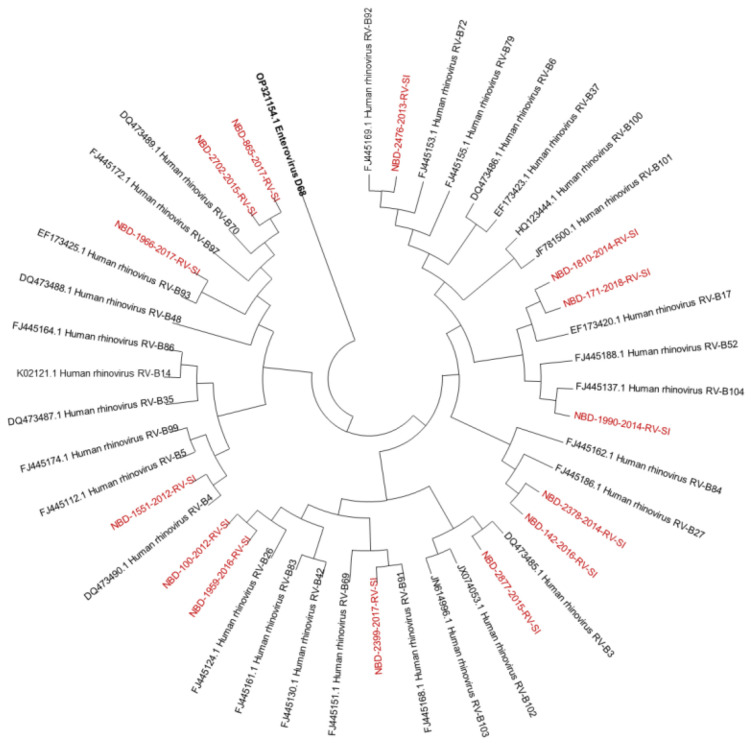
Phylogenetic tree (RV-B) constructed using the Neighbour-Joining algorithm, based on the VP4/VP2 region of RVs, including the selection of RVs genotyped in this study and RV-type reference strains defined by the *Picornaviridae* Study Group at ICTV [7]. Enterovirus EV-D68 (GenBank accession number: OP321154.1) was used as the outgroup. The RVs genotyped in this study are shown in red and labelled by the identifier NBD, followed by the laboratory protocol number, the collection year and the identifier RV-SI (NBD-PPPP-YYYY-RV-SI). All reference strains were retrieved from GenBank and are labelled by their GenBank accession numbers and RV type [30].

**Figure 3:**
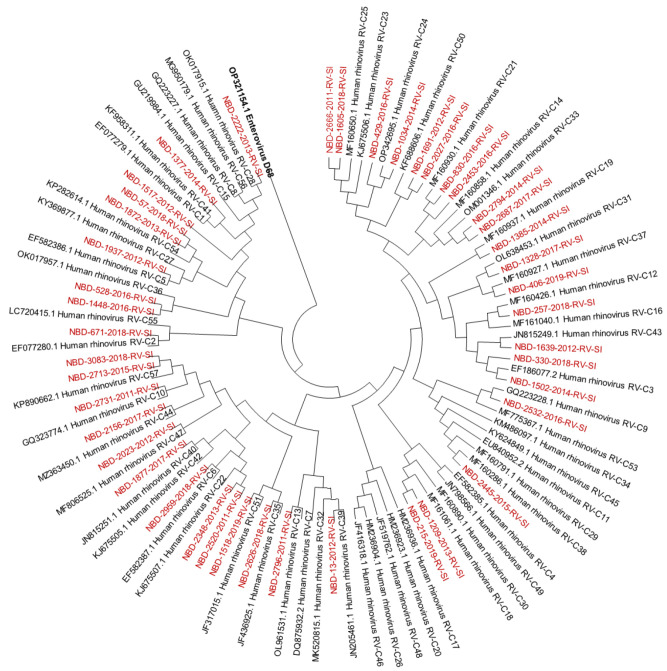
Phylogenetic tree (RV-C) constructed using the Neighbour-Joining algorithm, based on the VP4/VP2 region of RVs, including the selection of RVs genotyped in this study and RV-type reference strains defined by the *Picornaviridae* Study Group at ICTV [7]. Enterovirus EV-D68 (GenBank accession number: OP321154.1) was used as the outgroup. RVs genotyped in this study are shown in red and labelled by the identifier NBD, followed by the laboratory protocol number, the collection year and the identifier RV-SI (NBD-PPPP-YYYY-RV-SI). All reference strains were retrieved from GenBank and are labelled by their GenBank accession numbers and RV type [30].

Out of all known RV-A types, 91% (*n* = 73/80) were detected. Out of all known RV-B types, 41% (*n* = 13/32) were detected. Out of all known RV-C types, 82% (*n* = 47/57) were detected. The diversity of genotypes in species RV-A, RV-B and RV-C is presented in Figure 1, Figure 2 and Figure 3, showing phylogenetic trees constructed with a selection of sequences from RVs genotyped in this study and RV-type reference strains defined by the *Picornaviridae* Study Group at ICTV [7].

### 3.2. Seasonality of Rhinoviruses

**Figure 4:**
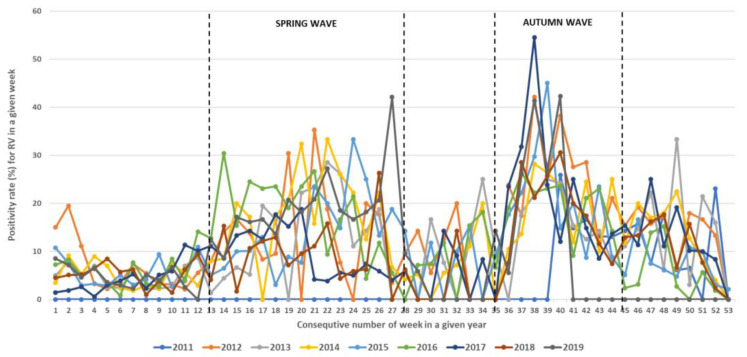
Seasonal circulation of RVs from 2011 to 2019 withspring and autumn waves. Black dashed lines show limits of spring waves (between weeks 13 and 28) and autumn waves (between weeks 35 and 45).

**Figure 5:**
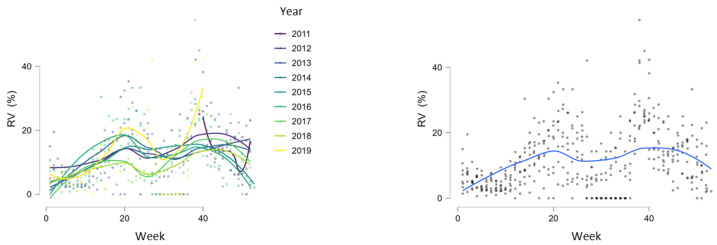
Generalised linear modelling of RV circulation with peaks of seasonal waves around weeks 20 and 40.

**Figure 6:**
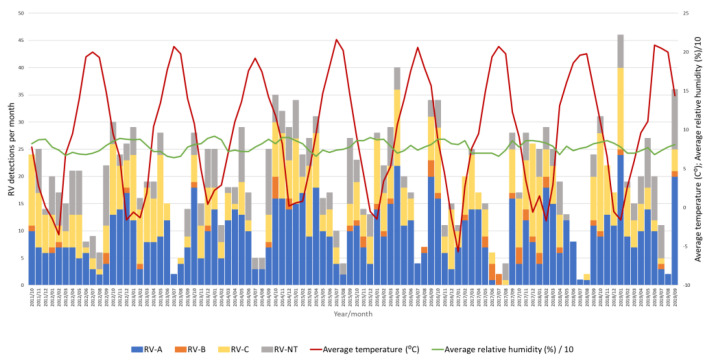
Number of RV-A, RV-B, RV-C and untyped RV (RV-NT) detections from week 40 in 2011 to week 39 in 2019 in relation to average temperature (°C) and average relative humidity (%).

During the study period, RVs circulated throughout the entire year but with different intensities, showing regular spring and autumn seasonal waves (Figure 4, Figure 5 and Figure 6). The spring wave was longer, spanning over approximately 15 weeks (between weeks 13 and 28), with a relatively flat peak (Figure 4). The autumn wave was shorter, spanning over approximately 10 weeks (between weeks 35 and 45), and was characterised by a sharper peak than in spring (Figure 4). The generalised linear modelling identified the spring peak as occurring around week 20 and the autumn peak as occurring around week 40 of each year (Figure 5).

Throughout the eight seasons, RV-A and RV-C constantly co-circulated with similar dynamics following seasonal alteration cycles, while RV-B did not show a clear circulation pattern and was detected sporadically in low proportions (Figure 6).

### 3.3. Impact of Meteorological Factors on Rhinovirus Circulation

A negative correlation between the average temperature and RV positivity was observed (Pearson’s r = −0.477, *p* < 0.05), meaning that an increase in RV positivity was observed when the average temperature dropped (Figure 6). On the contrary, there was a positive correlation between the average relative humidity and RV positivity (Pearson’s r = 0.357, *p* < 0.05), meaning that an increase in RV positivity was observed when the average relative humidity increased (Figure 6). However, a multiple regression analysis investigating the causality of these meteorological factors and RV positivity rates showed that only temperature had a statistically significant effect on RV circulation and positivity rates (*p* < 0.05), while relative humidity had no statistically significant effect on RV circulation and positivity rates (*p* = 0.27).

### 3.4. Variability in Rhinovirus Species and Types between and within Seasons

Throughout the study period, all RV species and 79% (*n* = 133) of all 169 known RV types were detected. The most predominant species was RV-A, followed by RV-C and RV-B (59%, 38% and 3%, respectively).

Out of the 80 known RV-A types, 91% (*n* = 73) were detected. Out of the 32 known RV-B types, 41% (*n* = 13) were detected. Out of the 57 known RV-C types, 82% (*n* = 47) were detected.

**Figure 7:**
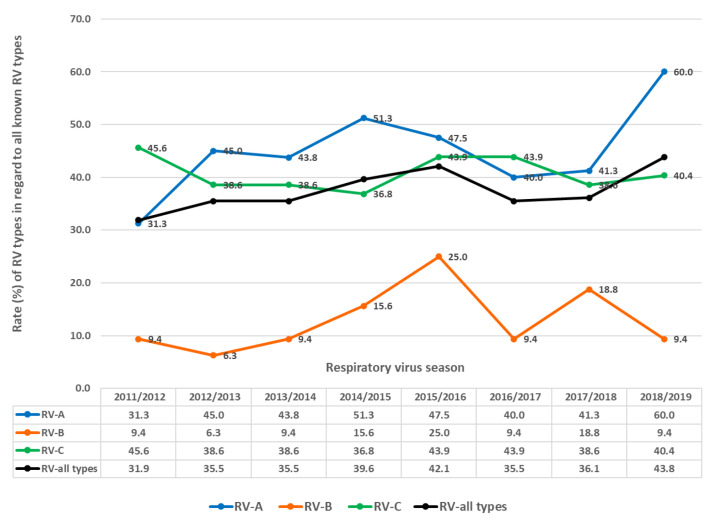
Rates (%) of different types regarding RV (all types), RV-A, RV-B and RV-C as the percentage of all known types of RV (all types), RV-A, RV-B and RV-C in investigated respiratory virus seasons.

**Figure 8:**
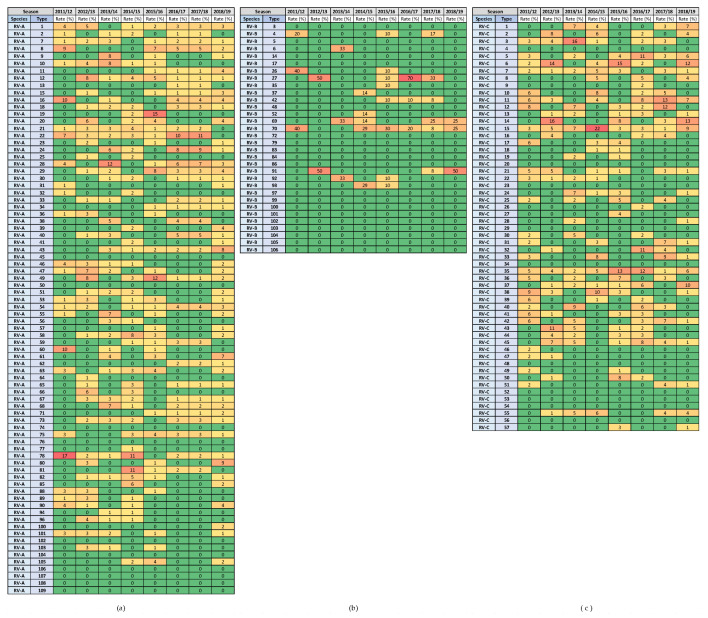
Heat diagrams of rates (%) of a given RV type as the percentage of all detected RV types of a given species in each respiratory virus season: (**a**) RV-A; (**b**) RV-B; and (**c**) RV-C. All known types of RV-A (80 types), RV-B (32 types) and RV-C (57 types) are present in the table. RV-B types were detected in low numbers. Red represents the highest rate, and green represents the lowest rate. A warm-to-cool colour scheme is used to show the decreasing rates.

In each season, multiple RV types circulated (Figure 7 and Figure 8), ranging from 32.0% to 43.8% of all known RV types per season (average = 37.5%, median = 35.8%). Between 31.3% and 60.0% of all known RV-A types were present per season (average 45.0%, median 44.0%). Between 6.3% and 25% of all known RV-B types were present per season (average = 12.9%, median = 9.4%). Between 36.8% and 45.6% of all known RV-C types were present per season (average = 40.8%, median = 44.0%).

The highest rates of detection for a single RV type per season were 17.4% for RV-A (RV-A78) and 21.5% for RV-C (RV-C15), while RV-B types were detected in low numbers. Extensive genotype variability was present, especially in RV-A species, followed by RV-C species (Figure 8). The same RV types were present in several consecutive or non-consecutive seasons (Figure 8). No RV-A was detected in all investigated seasons, but two were detected in seven out of eight seasons (RV-A54 and RV-A78). Additionally, RV-C15 and RV-C35 were detected in all investigated seasons, while RV-C7 was detected in seven out of eight seasons.

In the seasonal waves, multiple RV species and types co-circulated, and no single RV species or type was responsible for a seasonal peak or outbreak (Table 2). No statistically significant difference in RV genotype diversity in spring and autumn waves was confirmed for RV-A (Student’s t(14) = −1.266; *p* < 0.226) or RV-C (Student’s t(14) = −0.523; *p* < 0.609). On the contrary, a statistically significant difference in RV genotype diversity between spring and autumn waves was confirmed for RV-B (Student’s t(14) = 3.745; *p* < 0.002). In autumn waves, higher rates of RV-B types were co-circulating (mean = 7.8%; SD = 4.1) compared to spring waves (mean = 1.6%; SD = 2.4). Simultaneously, around 20% of all known RV-A genotypes co-circulated during the spring and autumn waves (spring wave mean = 21.9%, autumn wave mean = 19.1%) together with around 14% of all known RV-C genotypes (spring wave mean = 14.5%, autumn wave mean = 14.3%).

### 3.5. Variability in Rhinovirus Species and Types by Age Group

The ANOVA analysis of the rates (%) of different RV types in comparison to all known RV types showed that there was a substantial and statistically significant difference in the variability of RV types present in the age group of young children (from 0 to 3 years of age) in comparison to all other age groups (F(5, 30) = 102.925; *p* < 0.05), as shown in Table 3. Among children between 0 and 3 years of age, the mean rate of different RV types in comparison to all known RV types per season was 28.6% (SD = 3.5). In other age groups, the mean rate of RV types in comparison to all known RV types per season ranged from 3.4% to 7.9%. This phenomenon was confirmed for all RV types and for the types in species RV-A, RV-B and RV-C (Table 3).

**Figure 9:**
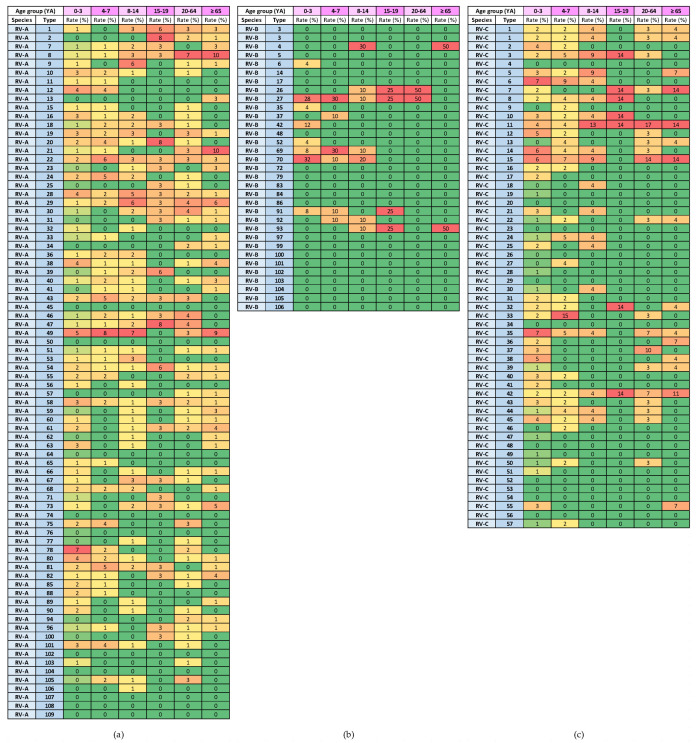
Heat diagrams of rates (%) of a given RV type as a percentage of all detected RV types of a given species in each age group (YA = years of age): (**a**) RV-A; (**b**) RV-B; and (**c**) RV-C. All known types of RV-A (80 types), RV-B (32 types) and RV-C (57 types) are present in the table. RV-B types were detected in low numbers. Red represents the highest rate, and green represents the lowest rate. A warm-to-cool colour scheme is used to show decreasing rates.

The heat diagrams in Figure 9 show the genotype distribution by age group. The genotypes of RV-A that were present in all age groups were RV-A8, RV-A22, RV-A28, RV-A29, RV-A54 and RV-A58 (6/80 types; 7.5%). The genotypes of RV-C that were present in all age groups were RV-C11 and RV-C42 (2/57 types; 3.5%).

### 3.6. Variability in Rhinovirus Species and Types in Patients with MAARIs and SARIs

The proportions of different RV species were similar in patients from primary healthcare clinics (patients with MAARIs) and from sentinel hospitals (patients with SARIs). In both groups of patients, RV-A predominated (66% and 58% in patients with MAARI and SARI, respectively), followed by RV-C (29% and 39% in patients with MAARI and SARI, respectively) and RV-B (5% and 3% in patients with MAARI and SARI, respectively).

A statistically significant difference in RV genotype diversity between patients with a SARI and MAARI was confirmed (Student’s t(14) = 17.309; *p* < 0.05). In patients with SARIs, higher rates of RV types in comparison to all known RV types were confirmed (mean = 35.4% of types, SD = 3.5) compared to patients with MAARIs (mean = 9.1% of types, SD = 2.4). This phenomenon was confirmed for all RV types and for the types in species RV-A, RV-B and RV-C (Table 4).

All RV types that were detected in the investigated population were found in patients with SARIs, while only some of these RV types were found in patients with MAARIs. No RV types were present exclusively in patients with MAARIs.

All RV-A types detected in our study were present in patients from hospitals, and 68% of these types were also detected in patients from primary healthcare clinics. RV-A types present only in patients with SARIs were 11, 13, 15, 21, 23, 32, 33, 34, 36, 39, 40, 41, 52, 57, 63, 64, 68, 71, 76, 88, 89 and 103. The only exception was RV-A106, which was detected in a single patient from a primary healthcare clinic.

All RV-B types detected in our study were present in patients from hospitals, and 46% of these types were also detected in patients from primary healthcare clinics. The RV-B types, present only in patients with SARIs, were 4, 6, 35, 37, 42, 52 and 92. 

All RV-C types detected in our study were present in patients from hospitals, and 59% of these types were also detected in patients from primary healthcare clinics. The RV-C types present only in patients with SARIs were 2, 6, 16, 17, 19, 21, 24, 25, 26, 28, 36, 40, 41, 42, 46, 47, 49 and 51.

## 4. Discussion

The present study is the first systematic analysis of RV molecular epidemiology in Slovenia and thus provides this missing scientific information at the national and regional levels. To our knowledge, it is also one of the largest studies on the molecular epidemiology of RVs in terms of study design, size and length [4]. We conducted an eight-year-long investigation on one of the largest study cohorts, with a total of 1480 genotyped RVs, and included patients from all age groups with respiratory infections of various severity who were examined in primary healthcare settings and in hospitals. We determined the RV genotype’s variability and seasonal circulation. Our study design, size and length allowed a statistically relevant investigation of genotyping data linked to demographical and meteorological data. Our study contributes complementary aspects and sound evidence-based knowledge to the regional and global understanding of RV molecular epidemiology.

Like most previous reports, our study confirmed year-round RV circulation with clear seasonal cycles, resulting in two seasonal waves with peaks in spring and autumn [4]. In our study, the spring wave was longer, with a relatively flat peak around week 20 of a given year (mid-May), in comparison to a shorter autumn wave with a sharper peak around week 40 (the beginning of October). Like many other reports, our study also observed a constant circulation of RV-A and RV-C, while RV-B did not show a clear circulation pattern [4,6]. However, in this context, several studies indicated the prevalence of RV-C in autumn and winter waves and a prevalence of RV-A in spring waves [18,35,36,37,38,39,40,41], while our study showed that in the investigated population, RV-A and RV-C both co-circulated throughout the year with similar dynamics and relatively constant proportions. In this study, the most predominant species was RV-A, followed by RV-C and RV-B, which could also reflect the fact that RV-A comprises a higher number of genotypes than RV-C and RV-B. Additionally, in our study, similar proportions of species were observed between patients from primary healthcare settings and patients from hospitals.

Our study, under many aspects, confirmed high levels of RV species and genotype variability and co-circulation in the investigated population. Many RV types identified through this investigation were present in several consecutive or non-consecutive years. A single RV type was not responsible for a single wave or peak. The highest rate of a single RV type per year was around 20%, indicating the co-circulation of various RV types, as previously described [4,6,42]. In our study, the RV genotype diversity and co-circulation were similar in spring and autumn waves for RV-A and RV-C, while RV-B showed greater genotype diversity in autumn waves.

When comparing age groups, a substantial and statistically significant difference in RV-type variability was identified among infants and young children (from 0 to 3 years of age) in comparison to all other age groups. However, caution is required when interpreting these datasets because, in this study, the paediatric patients outnumbered the patients from other age groups. Additionally, children, in comparison to adult patients, are more prone to be examined in a medical setting because the parents of small children consult clinicians more often, even in cases of milder clinical manifestations, such as the common cold. High levels of RV-type variability were also observed in patients examined in primary healthcare settings and in patients examined in hospitals. A statistically significant genotype diversity was observed in hospitalised patients compared to patients examined in primary healthcare settings. All RV types that were identified in this study were detected in hospitalised patients, and over half of these same RV types were also detected in patients from primary healthcare settings, while no RV type was detected exclusively in patients from primary healthcare settings. These results indicate that we should reject our initial hypothesis that RV-type variability might be higher in patients from primary healthcare clinics than in hospitalised patients. Consequently, we identified several RV types among the species RV-A, RV-B and RV-C that were present only in hospitalised patients, which could indicate the capability of these specific RV types to cause more severe respiratory infections. However, these results should be interpreted with caution because, in this study, patients with SARIs outnumbered patients with MAARIs, and more data should be collected and analysed before confirming such conclusions.

The present study provides evidence that many factors affect RV circulation and seasonality. It is important to understand the impact of these factors as they may differ between countries and regions. They could, therefore, explain some inconsistent or contradictory conclusions on RV circulation between different studies and should be considered for the effective national and regional planning of public health control measures aiming to lower the high socioeconomic burden of RVs.

Environmental factors, such as meteorological parameters, have an impact on RV circulation [4]. In this study, correlations between increased RV circulation, lower temperatures and higher humidity levels were identified. However, a statistically significant causal effect on increased RV circulation was confirmed by multiple regression only for lower temperatures. This result is consistent with other similar studies [4]. On the other hand, some previous studies also report the significant role of high humidity on increased RV circulation [43,44], contradicting studies that found increased RV circulation in periods with low humidity [18,45]. Understanding the effects of environmental factors on RV circulation is becoming even more important in the current era of climate change for the implementation of adequate public health measures to contain the burden of RV infections in the community.

The seasonal circulation of other respiratory viruses could also impact RV circulation, but this aspect was not investigated in this study. However, viral interference between respiratory viruses has been described previously [46].

Social factors could also impact RV circulation. Our data showed a regular and sharp increase in RV circulation between September and October, which coincides with the start of the school year in Slovenia but could also be facilitated by the seasonal drop in temperature and increase in humidity due to higher levels of rain in autumn. In Slovenia, most children aged between one and five years of age attend kindergartens, which have no formal holiday periods, so preschool children mingle all year round. Usually, the number of children enrolled in kindergartens drops over the summer because they go on vacation with their parents and siblings, but it can be assumed that this does not affect the circulation of RVs to a significant extent. Additionally, considering the school calendar in Slovenia, with four one-week breaks during the school year (winter, spring, autumn and Christmas/New Year breaks on weeks 8, 18, 44 and 52, respectively) and a 10-week summer holiday, these one-week breaks during the school year do not seem to significantly drive a reduction in RV circulation. The decrease in RV circulation starts even before the autumn break (the seasonal peak occurred around week 40, while the autumn break is on week 44). The spring seasonal wave was identified between weeks 13 and 28, and the spring break in week 18 seemingly did not significantly affect the circulation of RV in this period.

However, children, with combined high detection rates and high RV-type variability, remain a potentially significant driving force in RV circulation. This phenomenon could be, in part, a consequence of the fact that young children are generally less able or prone to strictly follow non-pharmaceutical preventive measures, such as hand washing, not touching their face, social distancing and wearing facial masks.

Slightly higher RV positivity rates were observed in male patients of all age groups, except for the age groups from 15 to 19 years of age and from 20 to 64 years of age, where a shift to slightly higher RV positivity rates in female patients was present. This could be due to the fact that in these age groups, more females are in close contact with children than males (mothers or grandmothers taking care of children; volunteers helping with childcare and education; and nurses, teachers and trainees in these professions, in which females predominate in Slovenia).

One of the motivations for our study was to also investigate whether there are specific RV genotypes that cause severe infections or outbreaks more often because this information could be useful in directing vaccine development in order to contain infections and reduce the socioeconomic burden of RVs. However, our results indicate that the selection of RV types for the development of a polyvalent RV vaccine could present a significant challenge due to the extensive RV genotype diversity and co-circulation. Attention could be given to RV genotypes that are observed to be present only in patients with SARIs, in multiple seasons or in multiple age groups. However, our results are not sufficient to drive such conclusions. Because of these limitations, the efforts to identify risk groups and develop alternative control measures and treatment strategies, at least in the short to middle term, should be continued.

There are additional limitations to our study to be considered. This is not a controlled study; therefore, an imbalance arose in the structure of the investigated patient cohort regarding the patients’ ages (children from 0 to 7 years of age represented a larger proportion of the studied population in hospitals) and regarding the clinical setting (hospitalised patients outnumbered the patients examined in primary healthcare settings), meaning that caution is required when interpreting the results. Many previous studies have similar limitations by focusing on exclusive population segments, such as children, adults and hospitalised or immunocompromised patients [4,6,12]. These facts compromise a comparison of results between different study groups.

Another limitation of our study that affects the formation of generalised conclusions on RV epidemiology is the national specificity of this study in terms of the demographic, meteorological and social factors that affect RV circulation, which might differ significantly between countries and regions.

One of the main focuses of this study was to describe the seasonality of RVs, which is affected by many factors. In this study, several factors were considered (patient age, disease severity and meteorological factors), and many aspects were discussed (social factors, such as school holidays and children’s behaviours). However, these are not exclusive, and they may vary regionally and globally, so biases should always be considered when interpreting results, especially in comparison to results from other studies.

Additionally, in our study, genotyping, although successful for a high proportion of the investigated RV population, was still not successful for all detected RVs, so 17% of these remained untyped. This might also be a source of bias in investigating the epidemiological features of RVs with high species and type variability as well as co-circulation [2,4,6,37,47].

## 5. Conclusions

The present study is the first systematic large-scale analysis of RV molecular epidemiology in Slovenia and provides missing scientific information on RVs at the national and regional levels. Due to its design, size and length, it also contributes new aspects and sound evidence-based information for global efforts in understanding RV’s molecular epidemiology. Our results describe RV circulation for the longest study period compared to published studies, which enables us to more precisely describe RV’s seasonality. Our results also emphasise the high variability and co-circulation of many RV genotypes under various aspects such as patient age, disease severity and meteorological factors. As most existing studies focus on exclusive segments of the population, predominantly investigating patients with clinical manifestations, in the future, studies should also include asymptomatic individuals and individuals with very mild clinical manifestations for a complete understanding of RV’s molecular epidemiology.

## Figures and Tables

**Table 1:** Distribution of RV-positive patients by age, gender (male, female) and patient source (SARI = sentinel hospital, MAARI = sentinel primary health care clinic).

**^1^ AG (^2^ YA)**	**1 (0–3)**		**2 (4–7)**		**3 (8–14)**		**4 (15–19)**		**5 (20–64)**		**6 (≥65)**	
	**^3^ * n***	**^4^ Rate (%)**	***n***	**Rate (%)**	***n***	**Rate (%)**	***n***	**Rate (%)**	***n***	**Rate (%)**	***n***	**Rate (%)**
Male	686	59.6	115	57.8	76	51.7	23	39.7	54	39.1	65	52.4
Female	465	40.4	84	42.2	71	48.3	35	60.3	84	60.9	59	47.6
SARI	1079	93.7	187	94.0	116	78.9	29	50.0	91	65.9	121	97.6
MAARI	72	6.3	12	6.0	31	21.1	29	50.0	47	34.1	3	2.4

^1^ AG = age group, ^2^ YA = years of age, ^3^ *n* = number of patients, ^4^ rates in (%).

**Table 2:** Mean rate (M, %) of RV genotypes in comparison to all known RV genotypes co-circulating during spring waves (weeks 13 to 28) and autumn waves (weeks 35 to 45).

	M (%)			
Seasonal Waves	RV (All Species)	RV-A	RV-B	RV-C
Spring waves	15.8	21.9	1.6	14.5
Autumn waves	15.4	19.1	7.8	14.3
*p*-value	0.706	0.226	0.002	0.609

*p*-value < 0.05 was considered statistically significant.

**Table 3:** Mean rate (M, %) of RV genotypes in comparison to all known RV genotypes in patients from different age groups.

	M (%)			
^1^ AG (^2^ YA)	RV (All Species)	RV-A	RV-B	RV-C
1 (0–3)	28.6	32.5	6.7	35.5
2 (4–7)	7.9	9.1	3.1	8.9
3 (8–14)	7.8	11.4	3.9	4.9
4 (15–19)	3.4	5.5	1.6	1.6
5 (20–64)	6.8	11.1	0.8	5.9
6 (≥65)	6.2	9.6	0.8	4.6

^1^ AG = age group, ^2^ YA = years of age.

**Table 4:** Mean rate (M, %) of RV genotypes in comparison to all known RV genotypes in patients with SARIs and MAARIs.

Severity of Infection	M (%)			
	RV (All Species)	RV-A	RV-B	RV-C
SARI	35.4	42.1	11.7	39.5
MAARI	9.1	12.5	2.7	7.9

## Data Availability

All data presented in this study are available on request from the corresponding author.
